# Diacylglycerol metabolism and homeostasis in fungal physiology

**DOI:** 10.1093/femsyr/foae036

**Published:** 2024-11-28

**Authors:** Sudipta Mondal, Biswajit Pal, Rajan Sankaranarayanan

**Affiliations:** CSIR – Centre for Cellular and Molecular Biology, Uppal Road, Hyderabad 50007, India; CSIR – Centre for Cellular and Molecular Biology, Uppal Road, Hyderabad 50007, India; CSIR – Centre for Cellular and Molecular Biology, Uppal Road, Hyderabad 50007, India; Academy of Scientific and Innovative Research (AcSIR), Ghaziabad 201002, India

**Keywords:** diacylglycerol, lipid metabolism, lipid second messenger, membrane contact site, acyl-chain diversity, organelle membranes, disco-interacting protein 2

## Abstract

Diacylglycerol (DAG) is a relatively simple and primitive form of lipid, which does not possess a phospholipid headgroup. Being a central metabolite of the lipid metabolism network, DAGs are omnipresent in all life forms. While the role of DAG has been established in membrane and storage lipid biogenesis, it can impart crucial physiological functions including membrane shapeshifting, regulation of membrane protein activity, and transduction of cellular signalling as a lipid-based secondary messenger. Besides, the chemical diversity of DAGs, due to fatty acyl chain composition, has been proposed to be the basis of its functional diversity. Therefore, cells must regulate DAG level at a spatio-temporal scale for homeostasis and adaptation. The vast network of eukaryotic lipid metabolism has been unravelled majorly by studying yeast models. Here, we review the current understanding and the emerging concepts in metabolic and functional aspects of DAG regulation in yeast. The implications can be extended to understand pathogenic fungi and mammalian counterparts as well as disease aetiology.

## Teaser

This review explores the metabolic and functional regulation of diacylglycerol (DAG) in yeast, highlighting its physiological roles, chemical diversity and implications in fungal pathogenesis.

## Introduction

Lipids are essential components of cells and are involved in multitudes of functions including the formation of membrane, metabolism, digestion, energy storage, cellular signalling, subcellular vesicle trafficking, and so on. The International Lipid Classification and Nomenclature Committee classified lipids into eight categories, e.g. fatty acyls, glycerolipids, glycerophospholipids, sphingolipids, sterol lipids, prenol lipids, saccharolipids, and polyketides (Fahy et al. 2005). Among these lipids, neutral diacylglycerols (DAGs) are the subclass of glycerolipids, which are of special interest considering their diverse roles in multiple metabolic processes and cellular signalling pathways. Impaired DAG metabolism has been implicated in several disorders in humans, such as defects in organ development and cell growth, and diseases including Alzheimer’s, cancer, diabetes, and immune system disorders (Carrasco and Merida 2007). Several studies on fungal pathogens have also confirmed the role of DAG metabolism in successful pathogenesis and virulence in plants and animals (Rhome and Del Poeta 2009). This underlines the importance of probing DAG metabolism to gain insights into fundamental cell biology and raise the possibility of targeting it for therapeutic interventions.

In this review, we begin by highlighting the physicochemical properties of DAG molecules, that are essential in creating platforms for multiple cellular functions. Next, we have illustrated the landscape of the DAG metabolism network, characterized in yeast and discussed its centrality to the fundamental physiology. Following this, we have discussed the emerging concepts of acyl-chain-based diversity in DAG pools and its debated role in cellular homeostasis and signalling. We have also provided an updated view of the upcoming field of subcellular DAG metabolism and an early insight into the mechanisms of DAG regulation at membrane contact sites (MCS). Finally, we have summarized reports that have revealed the impact of DAG-mediated processes on the growth and virulence of multiple free-living and pathogenic fungi.

## Structure and chemistry of DAGs – simple yet atypical

**Figure 1.**
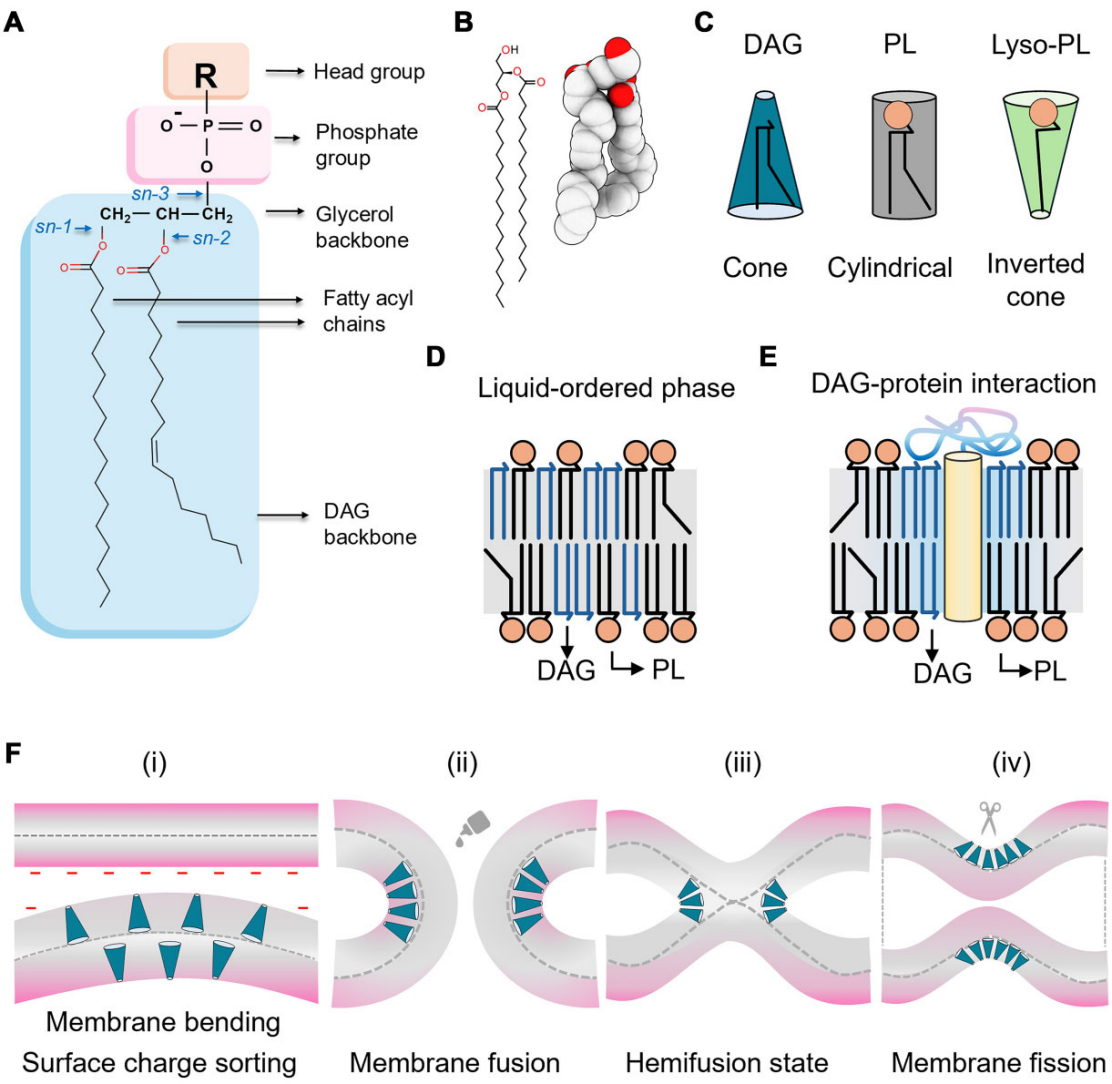
(A) Representative chemical structure of glycerophospholipid (PL) highlighting the core DAG backbone (in blue). The glycerol backbone is attached to representative saturated and unsaturated fatty acyl chains in the *sn-1* and *sn-2* positions, respectively. Chemical modifications on *sn-3* hydroxyl group (–OH) of DAG with phosphate group (pink) and other headgroups (R = choline, ethanolamine, serine, and inositol shown in orange) produce different classes of PLs, while attachment of a fatty acid to the *sn-3* position gives rise to triacylglycerols (TAGs). (B) Representative chemical structure of DAG molecule in stick (harbouring saturated fatty acyl chains) and space-filling three-dimensional model. (C) Graphical depiction of the conical shape of DAG molecules, along with PLs and lyso-PLs having cylindrical and inverted conical shape, respectively. (D) A model showing the phase behaviour of DAGs (in blue) in a typical lipid bilayer. (E) DAG-rich regions in lipid bilayer can facilitate specific lipid–protein interactions. DAGs are shown in blue colour and the transmembrane domain of the protein model is depicted as cylinder (yellow), with the extracellular region as ribbon (blue). (F) Visualization of different membrane topologies facilitated by DAG molecules (shown in blue conical shape). Transient accumulation of DAGs helps in membrane bending and neutralization (grey surface) of negative surface charge (red surface) (i). A higher level of DAG accumulation in the inner leaflet of the membrane bilayer facilitates negative curvature required for membrane fusion and hemifusion state (ii and iii), while negative curvature on the outer leaflet promotes membrane fission (iv). Glue and scissor icons in (ii) and (iv) indicate membrane topologies that lead to fusion and fission processes, respectively.

DAG is a simple glycerolipid comprising a glycerol moiety linked to two fatty acids through ester bonds at any two of the three positions, namely the *sn-1, sn-2*, and *sn-3* (Fig. 1A). DAG can be present in three different stereoisoforms – *sn-1,2; sn-2,3*; and *rac-1,3*. Although *sn-1,2* isoform is most prevalent and involved in all the known functions of DAG in cells, the other isoforms may possess yet unknown biological functions (Eichmann and Lass 2015). Different levels of unsaturation of fatty acyl chains add further diversity to the DAG pool (Fig. 1A and B), whereas different phospholipid headgroups along with the number and types of acyl chains give rise to different shapes in other glycerophospholipids (PL) (Fig. 1C). DAG possesses remarkable physicochemical properties (Israelachvili et al. 1980, Goni and Alonso 1999) and can flip-flop easily across transbilayer membranes without the help of proteins (Hamilton et al. 1991, Contreras et al. 2010). Because of the amphipathic nature, PLs form membrane bilayers where the hydrophobic lipid regions (fatty acid tails) are shielded from the extracellular medium, while the polar headgroups are oriented towards the external aqueous environment. In contrast, the distinct shape and chemistry of DAG bestows this molecule with a set of unique functions in the membrane. Since there are excellent reviews available on this topic (Sprong et al. 2001, Carrasco and Merida 2007, Almena and Merida 2011, Eichmann and Lass 2015), we offer a brief summary to provide an overview.

### Membrane curvature

The space requirements of the lipid headgroup and hydrophobic tail determine the geometric shape of the lipid, which affects the curvature and morphology of the lipid membrane (Fig. 1C). The headgroup of DAG comprises of only a hydroxyl group of glycerol moiety, which is much smaller than other lipid headgroups. This enables DAGs to create negative curvature in the membrane. The lack of charge on the headgroup of DAGs causes unstable asymmetric patches in membrane bilayers when they are concentrated on nanometer or micrometer-scale membrane sections. Localized accumulation of DAGs induces an intermediate topology in the membrane, enhancing curvature to reduce the physical tension (Fig. 1Fi). Such DAG-mediated membrane curvatures can create prefusion or hemifusion states, which are prerequisite for membrane fusion (Carrasco and Merida 2007, Starr and Fratti 2019).

### Membrane fusion and fission

DAG accumulation on the outer leaflet of the membrane can induce constriction, thus altering the membrane topology, and subsequently, the fission process. In contrast, DAGs at the luminal side of the membrane support the hemifusion state to facilitate fusion (Fig. 1Fii–iv). DAG has been shown to accumulate on the vertex area of the docked (prefusion state) vacuole membrane to support the highly curved surface and help attain the hemifusion state, which eventually leads to membrane fusion (Starr and Fratti 2019). Similarly, an *in vitro* study has shown that vesicles devoid of small headgroup neutral lipid, like DAG, can achieve a docked state with the help of anchor proteins, but they are unable to fuse due to problems in lipid rearrangement (Zick et al. 2014). In a more physiological context, studies have provided evidence that DAG production from phosphatidylinositol-4,5-bisphosphate (PIP2) helps in vacuole fusion (Jun et al. 2004), while DAG to phosphatidic acid (PA) conversion may help in reducing vacuole fusion in yeast (Miner et al. 2017).

While DAG’s role in membrane fusion has been known for long, its role in fission has been less studied in the field. However, both fusion and fission of membrane-bound organelles are crucial for physiological functions. A study on peroxisome fission of yeast *Yarrowia lipolytica* has provided a mechanistic view of the process (Guo et al. 2007). The work suggests that the DAG produced in the inner leaflet of the peroxisomal membrane flips towards the outer leaflet at the maturation stage, resulting in membrane bending and subsequent recruitment of protein complexes required for fission. A few recent studies in mammalian systems have also established the role of DAG in Golgi (Bossard et al. 2007, Malhotra and Campelo 2011) and mitochondria (Huang et al. 2011, Baba et al. 2014) fission by facilitating the recruitment of membrane fission machineries such as dynamin-related proteins.

### Phase behaviour of membrane

Lipids in the membrane can form small domains (on the nanometer to micrometer scale) with distinct physical properties. These domains on the membrane have been shown to influence different physiological processes and protein functions. Such lipid domains are maintained by a proper balance between liquid ordered (*L_o_*) and liquid disordered (*L_d_*) phases: a crucial topic in membrane biology that has been extensively reviewed (Levental et al. 2020, Levental and Lyman 2023) (Fig. 1D). Recently, lipid domains have been probed *in vivo* using vacuoles of living yeast cells (Toulmay and Prinz 2013, Rayermann et al. 2017). Interestingly, DAGs have been reported to likely be enriched in the *L_o_* phase of yeast vacuole membrane (Ganesan et al. 2015, 2019). Using protein-based DAG sensors, the study has shown that DAG-enriched microdomains on vacuoles are excluded from regions demarcated by vacuolar-ATPase V0 domain, Vph1, a marker for *L_d_*domains (Toulmay and Prinz 2013). Also, several *in vitro* biophysical experiments have revealed that DAG incorporation in the model membrane with varying concentrations can induce a transition from bilayer (lamellar phase) to nonbilayer (hexagonal phase) membrane structure (Cooke and Deserno 2006, Contreras et al. 2010). Due to its smaller headgroup, DAG accumulation in micro- or nanodomains can expose hydrophobic regions of neighbouring lipids. Thus, DAG abundance in the membrane affects the activity of membrane-integrated or membrane-attached proteins by influencing the hydrophobic interactions between proteins and membrane lipids (Lentz et al. 2000) (Fig. 1E). However, further studies are needed for a detailed understanding of the physiological significance of DAG-based modulation in membrane phase behaviour.

## Navigating through the metabolic network of DAG

DAG is present at the crossroad of membrane and storage lipid synthesis. The convergence of multiple lipid metabolism routes into DAG production underscores its importance in physiological processes. On the other hand, catabolism of DAG balances cell growth and energy storage.

### DAG anabolism: convergence of multiple metabolic routes

**Figure 2.**
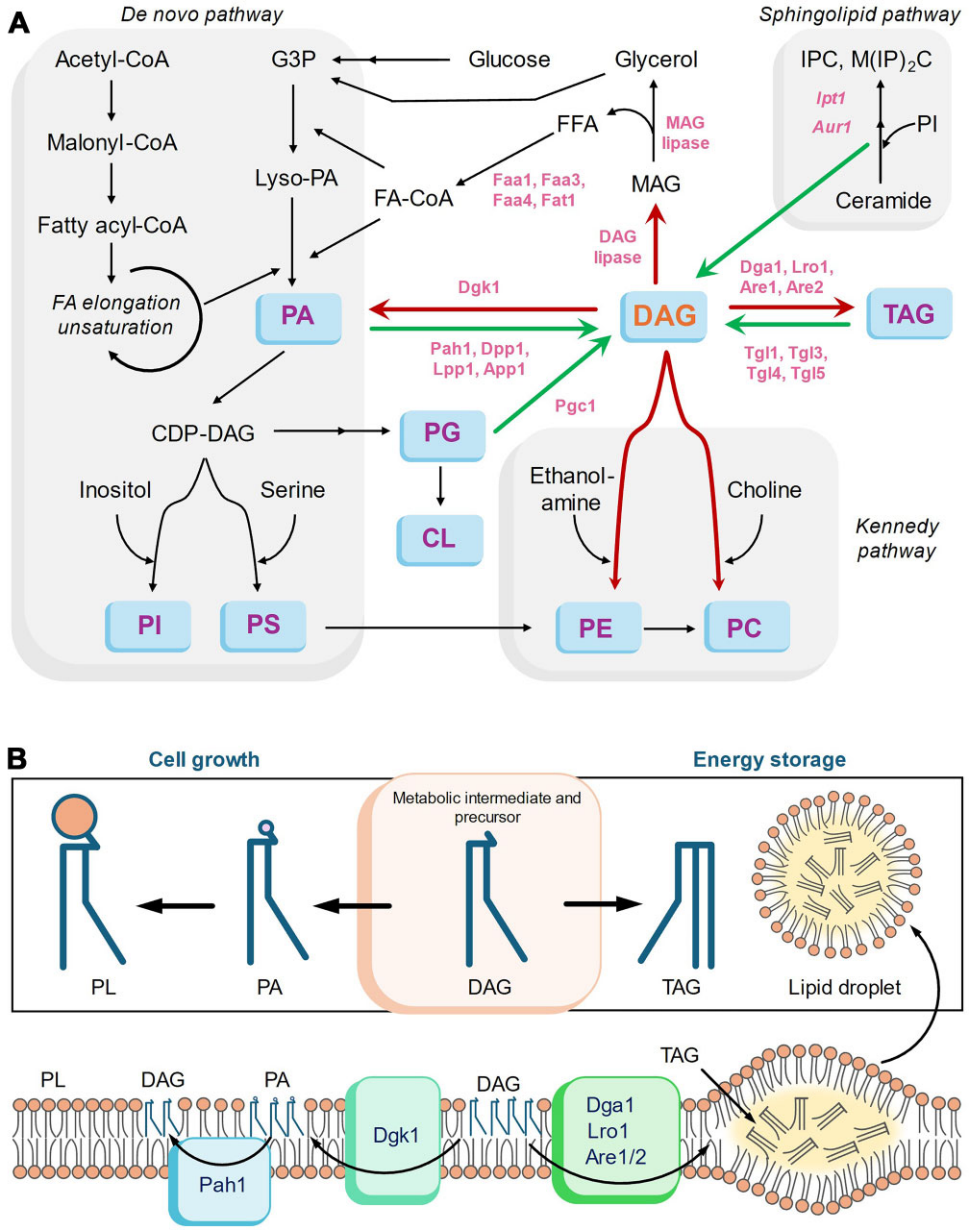
(A) Metabolic pathways for anabolism and catabolism of DAG in model yeast *Saccharomyces cerevisiae*. Major lipid metabolites are highlighted in blue boxes and the enzymes are represented in pink font. Green and red arrows indicate multiple anabolic and catabolic pathways, respectively, that converge on DAG. CDP-DAG, Cytidine diphosphate diacylglycerol; CoA, Coenzyme A; Lyso-PA, Lysophosphatidic acid; Inositol phosphoceramide, IPC; and Mannosyl-di-(inositol­phosphoryl)-ceramide, M(IP)_2_C. (B) DAG as the central metabolite at the crossroad of cell growth (membrane biogenesis) and energy storage (TAG-based LD production). Enzymes involved in the processes are indicated in blue.

DAG can be synthesized through the *de novo* lipid synthesis pathway and can also be generated from other lipid classes (Fig. 2A). In the *de novo* pathway, fatty acids are esterified with glycerol-3-phosphate to produce PAs (Henry et al. 2012). DAG is synthesized from PA through the hydrolysis of the attached phosphate group by PA phosphatase enzymes. Four PA phosphatases were identified in yeast encoded by *PAH1, APP1, DPP1*, and *LPP1* (Han et al. 2006, Carman 2019). However, only Pah1 is specific to PA, which produces bulk DAG species as precursors for membrane PL synthesis. The other three PA phosphatases have been shown to act on other substrates as well and are speculated to be involved in signalling processes (Pascual and Carman 2013). Another major route of DAG production is hydrolysis of triacylglycerol (TAG) by enzymes called TAG lipases. There are multiple TAG lipases encoded by *TGL1, TGL3, TGL4*, and *TGL5*, which are mainly localized to lipid droplets (LDs; Kurat et al. 2006, Kohlwein 2010). During the early log phase, TAG lipolysis provides a steady-state DAG pool, which is utilized mainly for PL synthesis via the Kennedy pathway (for phosphatidylcholine, PC and phosphatidylethanolamine, PE) and CDP-DAG pathway (for phosphatidylinositol, PI and phosphatidylserine, PS) to support cell growth (Sorger and Daum 2003, Ouahoud et al. 2018) (Fig. 2A).

Different PL and sphingolipid, another major class of membrane lipid, also serve as the physiological source of the DAG pool. For instance, a particular DAG pool is generated from the PI pool during the synthesis of complex sphingolipids in yeast (Cerbon et al. 2005, Ganesan et al. 2019). A recent study has revealed that turnover of phosphatidylglycerol (PG) lipids in mitochondria by a putative phosphodiesterase, Pgc1, results in DAG production (Simockova et al. 2008). However, the fate and impact of this DAG pool are unknown. Similarly, phospholipase-based hydrolysis of PC has been proposed as a source of DAG, while the identity of the enzyme in yeast is still unknown (Marini et al. 1996).

### DAG catabolism: the balance between cell growth and energy storage

After *de novo* synthesis, DAGs are generally consumed to produce PLs such as PC, PA, PI, PE, and PS for membrane generation and TAGs for lipid storage (Fig. 2B). In the first step, DAG is phosphorylated by DAG kinase (Dgk1) to produce PA, which is further channelled for PL synthesis via multiple other enzymes. Dgk1 is the only known DAG kinase in yeast (Han et al. 2008a) and is structurally different from mammalian DAG kinases. It is also mechanistically unique as it synthesizes PA using cytidine triphosphate instead of adenosine triphosphate (Han et al. 2008b). Dgk1 is an integral membrane protein that has been found to be associated with the endoplasmic reticulum (ER) (Kosodo et al. 2001) and vacuole membrane fractions (Miner et al. 2017). The function of Dgk1 in yeast is important during growth resumption from the stationary phase as it helps channel DAG generated from TAG hydrolysis towards membrane synthesis (Fakas et al. 2011).

In yeast, there are four enzymes known for the synthesis of TAG using DAG as a substrate, i.e. Dga1, Lro1, Are1, and Are2 (Czabany et al. 2007). Dga1 and Lro1 are the major contributors of TAG synthesis (∼80%–90%), where they acylate DAG to produce TAG, utilizing fatty acyl-CoA and PC as acyl group donors, respectively. Dga1 is responsible for TAG synthesis mainly in the stationary phase and contributes less during the log phase growth (Oelkers et al. 2002). On the contrary, Lro1 expression has not been able to incorporate oleate into TAG in the stationary phase and active in the log phase (Oelkers et al. 2000). These observations are also supported by the transcriptional upregulation of Dga1 and Lro1 in their respective growth phases (Dahlqvist et al. 2000, Gasch et al. 2000). Two other isoenzymes with acyl-CoA: sterol acyltransferase activity, Are1 and Are2, are also involved in DAG acylation at a very low level, mainly in the stationary phase (Yang et al. 1996). The deletion of all four genes (*dga1, lro1, are1*, and *are2*) results in an almost complete depletion of storage lipids and a massive DAG accumulation (Sandager et al. 2002, Sorger and Daum 2003). These studies have also shown that bulk DAG channelling for storage lipid synthesis is not essential in yeast but crucial for growth revival and long-term survival in the stationary phase. Based on the growth phases of fungi, cells deal with a trade-off between cell growth and energy storage, and therefore spatio-temporal regulation of DAG’s metabolic fate is crucial for maintaining optimal physiology (Fig. 2B).

## Regulation of diverse DAG lipid species

**Figure 3.**
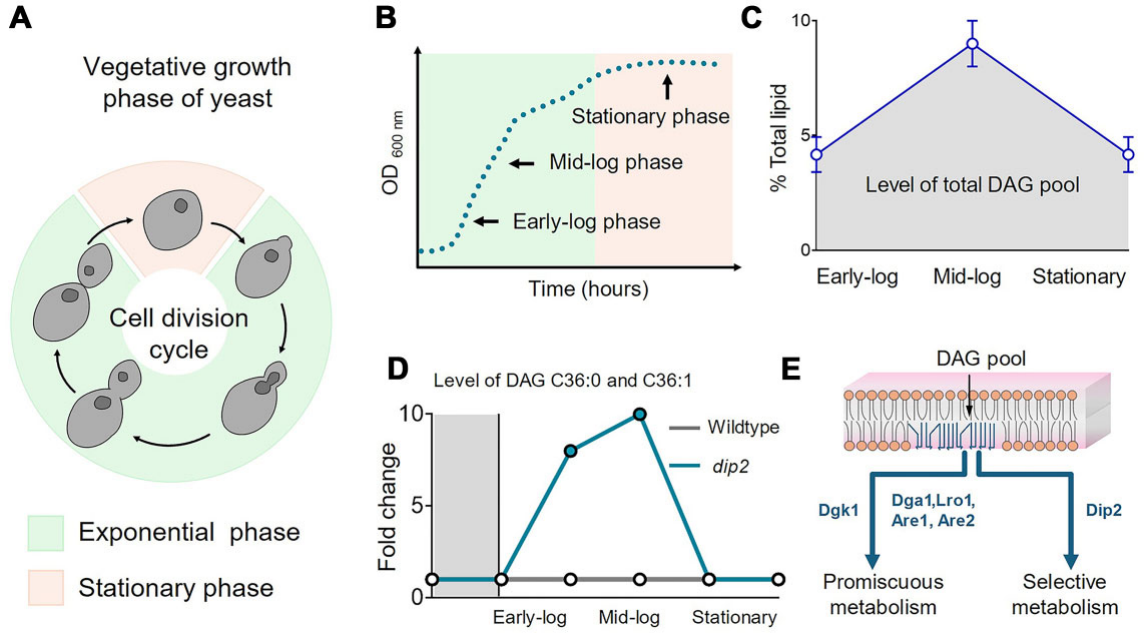
(A and B) A graphical depiction of the cell division cycle and different growth phases of yeast. (C) Alteration in the level of total DAG pool across the growth phases of yeast cells. Findings from Casanovas et al. (2015) and Mondal et al. (2022), have been adapted and presented as a simplified graph to visualize the dynamics in DAG levels (% relative to total lipid content). (D) A representative graph (adapted from Mondal et al. 2022) showing an accumulation of selective DAG species (C36:0 and C36:1) in the absence of Dip2/Cmr2 enzyme (*dip2*) in yeast. The model suggests that Dip2 is required to regulate the selective DAG pool during the early and mid-log phases of yeast growth. (E) A model showing the metabolic divide between selective metabolism of specific DAG species and promiscuous metabolism of bulk DAG pool, involved in different physiological functions. The genes currently known to be involved in these pathways are mentioned, while further research is needed to identify additional molecular players.

Rewiring of the lipid metabolism network happens throughout the growth phases of yeast. A reciprocal dynamics of membrane and storage lipid coincides with yeast growth phases and the DAG level is central to this (Fig. 3A and B). Metabolic radiolabelling and lipidomic analysis studies suggest that while the overall DAG levels are tightly regulated to remain nearly constant, a slight increase can be observed during the early and mid-log phases (Kohlwein et al. 2013, Casanovas et al. 2015) (Fig. 3C). In contrast, a ‘metabolic reset’ happens when cells enter the stationary phase and the increased synthesis of TAG depletes the DAG level (Casanovas et al. 2015, Mondal et al. 2022). Furthermore, environmental conditions and external stresses (e.g. ER stress) also cause alteration in TAG formation (Taylor and Parks 1979, Fei et al. 2009, Henry et al. 2012), which may indicate its possible link to DAG regulation.

With the advent of mass spectrometry-based lipid analysis, it has become more evident that eukaryotes possess a remarkable diversity of DAG species, varying in acyl chain length and the level of unsaturation (Hodgkin et al. 1998, Wakelam 1998, Raghu 2020, Schuhmacher et al. 2020). Even a relatively simple model eukaryote like yeast shows significant chain length diversity (Hofbauer et al. 2014) with a relatively simpler set, mostly composed of C16:0, C16:1, C18:0, and C18:1 acyl chains (Ejsing et al. 2009). Similarly, a comprehensive lipidome analysis of *Saccharomyces cerevisiae* identified around 193 different molecular species of DAGs (Danne-Rasche et al. 2020). It has been speculated for long time in the field that these DAG subspecies may govern distinct physiological processes. Recently, our study has identified a conserved eukaryotic protein, DISCO-interacting protein 2 (Dip2; encoded by *DIP2*/*CMR2*/*YOR093C* gene), that regulates a chemically distinct pool of DAG (C36:0; 18:0/18:0 and C36:1; 18:0/18:1) in *S. cerevisiae* (Mondal et al. 2022). We have shown that Dip2 is involved in facilitating the conversion of such DAG subpopulation to TAG species without affecting the rest of the lipidome. Interestingly, the abundant DAG species such as C16:1/18:1 (34:2), C16:1/16:1 (32:2), C16:0/16:1 (32:1), and C16:0/18:1 (34:1), which are also reported in other lipidomics studies (Ejsing et al. 2009), are not under Dip2-based regulation. Our work further suggests a growth phase-specific regulation of the DAG subpopulation, which is governed by Dip2 expression (Mondal et al. 2022) (Fig. 3D). In a similar line, DAG containing monounsaturated fatty acids are found to be almost as abundant as those containing saturated fatty acyl chains in the log phase while monounsaturated fatty acid-containing DAG species become dominant during the stationary phase (Casanovas et al. 2015). Casanovas et al. (2015) have explained that such profile of DAG in the log phase probably reflects a pool of DAG derived from PI, which serves as a precursor for the synthesis of complex sphingolipids in the Golgi during active growth.

The profile of DAG species depends on the specificity of enzymes involved in DAG metabolism. The canonical DAG metabolizing enzymes, including DAG kinases and DAG acyltransferase, are promiscuous mainly towards DAG substrates while possessing a certain degree of specificity for fatty acyl substrates. For instance, Dga1, Are1, and Are2 use fatty acyl-CoA as a substrate with different acyl chain length specificities *in vitro* (Oelkers et al. 2002). Dga1 prefers oleic acid (C18:1) and palmitoleic acid (C16:1) as substrates more than other fatty acids like myristoleic acid (C14:1), stearic acid (C18:0), arachidonic acid (C20:4), and linoleic acid (C18:2). On the other hand, Are1 and Are2 readily incorporate linoleoyl-CoA, while utilize palmitoyl-CoA less efficiently (Yang et al. 1996). It is important to note that polyunsaturated fatty acids like arachidonic and linoleic acid, used in the aforementioned *in vitro* experiments, are not naturally present in *S. cerevisiae*. However, these fatty acids are known to be readily absorbed from the growth medium and incorporated into the membrane (Ferreira et al. 2011). Lro1, being a transacylase, uses PLs as fatty acyl donors and DAG as an acyl acceptor to produce TAGs (Czabany et al. 2007). Lro1 utilizes the *sn-2* acyl group of PL for the acylation of DAG, while the headgroup of PLs influences the acylation step (Dahlqvist et al. 2000). Previous studies have shown that dioleoyl-PE (C18:1/18:1 PE) is preferred substrate compared to dioleoyl-PC (C18:1/18:1 PC) for Lro1 enzyme. These observations suggest that the nature of acyl donor species, the acyl chain composition of acceptor DAG molecules, and the headgroups PL acyl donor strongly influence the specificity and rate of acyl transfer during DAG metabolism. However, knockout of these DAG metabolizing enzymes results in the accumulation of bulk DAG pool (Oelkers et al. 2000, 2002, Fakas et al. 2011, Mora et al. 2012, Rockenfeller et al. 2018). Surprisingly, unlike the *dip2* knockout yeast, which accumulates a specific DAG subpopulation, the accumulation of bulk DAG pool in *dga1* or *lro1* or *dgk1* knockouts does not lead to unfolded protein response or osmoadaptation defects (Mondal et al. 2022). In contrast, bulk DAG accumulation results in global endomembrane defects such as loss of Golgi, bulging of ER membrane, and lipotoxic cell death (Rockenfeller et al. 2018, Li et al. 2020). These observations suggest a potential model involving two functionally distinct DAG populations, *viz*., a bulk DAG pool primarily utilized mainly for lipid bilayer production and energy storage, and a context-dependent DAG subpopulation required for other subcellular functions (Fig. 3E).

## Subcellular locations of DAG metabolism

**Figure 4.**
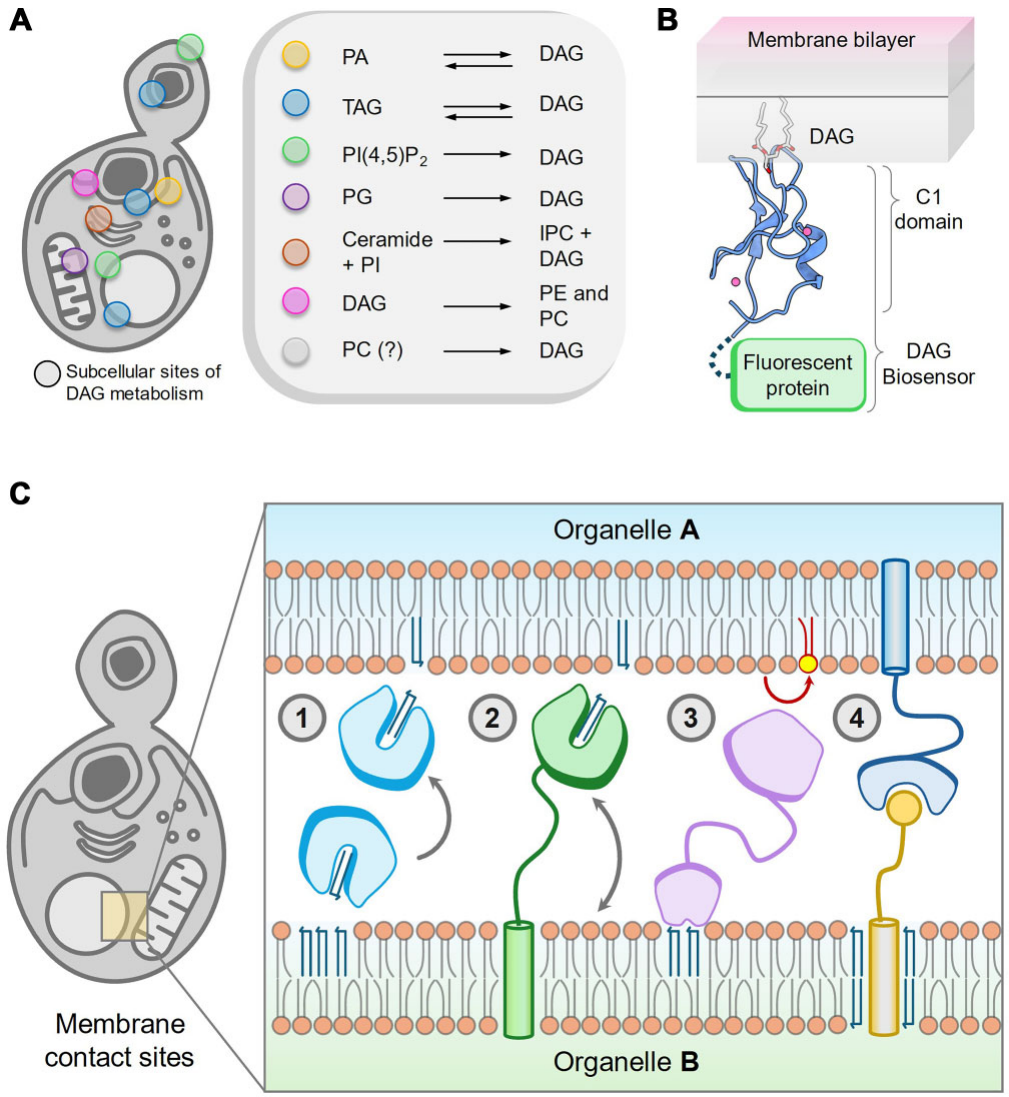
(A) Different subcellular locations of yeast cells can harbour metabolically diverse DAG pools, which are involved in different physiological processes. (B) A graphical representation of C1-domain-based DAG biosensor. C1-domain that recognizes glycerol moiety of DAG, can be tagged with fluorescence proteins to track DAG pool in live cells. (C) A graphical representation depicting different putative modes of DAG regulation at organellar contact sites using hypothetical protein models. (*1* and *2*) DAG transport and exchange between organellar membrane via soluble (*1*) and membrane-bound proteins (*2*). (*3*) Localized DAG metabolism at organellar contact sites by residing enzymes. (*4*) DAG-dependent sorting and functionalization of membrane-bound proteins to establish or maintain contact site architecture.

DAG metabolism is known to occur at multiple subcellular sites, such as ER, vacuoles, LDs, Golgi apparatus, plasma membrane (PM), and so on. (Fig. 4A). Since DAG is an intermediate in the anabolism and catabolism of diverse lipid products, cells must segregate different pools of DAG involved in different metabolic pathways. Hence, different compartments of cells have been employed as distinct sites for DAG metabolism. *De novo* synthesis of DAG takes place in the ER membrane and is further converted to TAG by ER-resident enzymes, Dga1, Lro1, Are1, and Are2. Studies have identified that the ER, ER-associated with nuclear envelope (Adeyo et al. 2011, Barbosa et al. 2015), and nuclear ER–vacuolar junctions (Hariri et al. 2018) are the site of DAG-to-TAG production. Although the steady-state level of DAG in ER is very low, deletion of these enzymes leads to the accumulation of bulk DAG pool in ER. The level of DAG accumulation is more severe during starvation and leads to cellular problems like defects in ER–Golgi vesicular transport, ER structure defect and inhibition of autophagy (Li et al. 2020). Such aberrant accumulation of DAG in ER can also lead to severe lipotoxicity and cell death, as demonstrated by genetic knockout studies of *dga1* and *lro1* in both *S. cerevisiae* and *Schizosaccharomyces pombe* models (Zhang et al. 2003, Rockenfeller et al. 2018).

Based on several microscopy-based studies on the localization of DAG biosensors (e.g. C1 domain of mammalian protein kinase C tagged with fluorescent proteins) (Fig. 4B), major DAG pools are found in vacuolar membrane and the site of polarized growth, i.e. PM of nascent bud site and shmoo projection during yeast mating (Ganesan et al. 2019). The DAG pool on the vacuole is enriched as punctate structures, which are possibly liquid-ordered phase membrane and closely associated with LDs. TAG lipolysis has been found to be the major source of these vacuolar and polarized PM DAG pools. On the other hand, DAG generated through inositol phosphoceramide (IPC) pathway in the Golgi also contributes to this polarized DAG pool (Ganesan et al. 2019). This subpopulation of DAG in Golgi has been shown to be crucial for vesicular trafficking from Golgi (Kearns et al. 1997). However, this approach can only detect the cytosol-facing and relatively high level of DAG pool on the organelle membrane. As a result, the relatively lower levels of DAG pools from the ER, Golgi, mitochondria, and other organelles are not detectable with these DAG sensors.

Some recent studies have tackled the limitation of soluble DAG sensors by targeting them to specific compartments like ER (Choudhary et al. 2018) and inner nuclear membrane (Romanauska and Kohler 2018) to study the dynamics and physiological relevance of spatially distinct DAG pools. ER-related DAG pool accumulates at the site of LD biogenesis (Choudhary et al. 2018), which is also found to be enriched with proteins involved in peroxisome formation (Joshi et al. 2018). This suggests that the DAG-enriched subdomains of ER serve as the site for both LD and peroxisome biogenesis. Interestingly, another major DAG pool has been identified on the inner nuclear membrane, utilized for nuclear LD biogenesis in yeast (Romanauska and Kohler 2018, Barbosa et al. 2019, Foo et al. 2023) by targeting the DAG sensor inside the nucleus. The inner nuclear DAGs are present throughout the membrane and possibly serve as a precursor for nuclear LD formation by Lro1 enzyme. This metabolic utilization of the DAG pool at the inner nuclear membrane is crucial for sustaining nutrient starvation and maintaining nuclear morphology (Barbosa et al. 2019). These observations also suggest that tracking DAG pools in other organelles utilizing similar techniques may shed light on many interesting aspects. In addition to different organellar membranes, recent studies have also demonstrated that the distribution of DAGs can be asymmetrical across the outer and inner leaflets of the membranes, depending on the surrounding lipid environment (Ueda et al. 2013, 2014).

## DAG metabolism at organellar contact sites

Studies from the last decade have highlighted the fact that the organelles are highly interconnected via special structures, named as ‘membrane contact sites’. MCS is classically defined as areas of close apposition between the membranes of two organelles (Scorrano et al. 2019) (Fig. 4C). As MCS are further characterized, it is increasingly evident that these contact sites function as crucibles of lipid metabolism and homeostasis (Vance 1990, Xu and Huang 2020). Recent studies have identified potential protein players that may operate at contact sites to modulate DAG levels across organelles through various mechanisms (A schematic has been shown in Fig. 4C). A recent example came from our study, where we have shown the presence of a selective DAG metabolizing protein, Dip2, at the mitochondria–vacuole contact sites in yeast. This raises the possibility of MCS-specific DAG metabolism, which could impact subcellular physiology by regulating organellar crosstalk and dynamics (Mondal et al. 2022). Similarly, a few recent studies have implied the metabolism of bulk DAGs at ER–LD contact sites. Ice2, an ER membrane-bound protein, has been proposed to work at the interface of ER and LDs. It helps in channelling LD-derived DAG (generated via TAG lipolysis) to ER for the production of membrane lipids (Markgraf et al. 2014). On the other hand, excess DAG level at ER–LD biogenesis sites decrease the chance of LD budding by promoting ER membrane-embedded states of LD (Choudhary et al. 2018). Fat storage-inducing transmembrane proteins 2 (Fit2) homologs, Scs3, and Yft2, have been shown to localize at ER–LD sites and impact LD budding. The ability of Fit2 to bind DAG *in vitro* leads to the proposition that it regulates DAG level at the ER–LD biogenesis site for efficient LD production and ER homeostasis (Gross et al. 2011, Choudhary et al. 2018, Becuwe et al. 2020).

During growth resumption from the quiescent stationary phase, DAG-rich structures are observed at vacuole–LD contact sites, where TAG lipolysis takes place to supply DAG as PL precursor (Ganesan et al. 2019). Proteomics of this DAG-rich membrane subphase has identified the presence of several different organelle contact site proteins, suggesting a metabolic hub to channelize DAG as raw material for membrane proliferation during the early growth phase (Ganesan et al. 2020). Proteins associated with different ER contact sites, such as Gem1 and Mdm10 of ER–mitochondria contact site, Vps13 of ER–vacuole, mitochondria–vacuole contact site, Ist2 and tricalbins (Tcb1–Tcb3) of ER–PM, Pex30 of ER–peroxisome contact site, have been found to be enriched in such DAG rich membrane fractions, opening new avenues for further investigations.

At ER–PM contact sites, extended-synaptotagmin (E-Syt), a lipid transporter protein, exchanges DAG to regulate cellular signalling in mammals (Saheki et al. 2016). Yeast homolog of E-Syt, Tcb1–Tcb3, are reported to be localized at ER–PM contact sites and functions to maintain PM integrity during stress conditions like heat (Collado et al. 2019, Thomas et al. 2022). Studies have revealed that Tcb induces positive curvature on cortical ER at the ER–PM contact site and genetically interacts with the Rim101 pathway (Hoffmann et al. 2019), which has previously been shown to be activated by elevated DAG levels (Rockenfeller et al. 2018). These observations raise the possibility that Tcb may function as DAG exchangers at ER–PM contact sites of yeast. Similarly, a mammalian ceramide transfer protein (CERT) has been shown to transfer ceramide from ER to Golgi at the ER–Trans–Golgi network contact site and can extract DAG, which is structurally similar to ceramide albeit with less efficiency (Hanada et al. 2003). Although CERT homolog is absent in fungi, recently identified ceramide transporters in yeast such as Svf1 and Nvj2 are shown to work at ER–Golgi contact sites (Liu et al. 2017, Limar et al. 2023). Since Golgi is a major site of DAG production via the sphingolipid biosynthesis pathway, yet unknown proteins might function as DAG transporters at the ER–Golgi contact sites.

Lipid metabolism at MCS is a rapidly evolving field, extending our understanding on cellular metabolism and physiology. In this context, the most crucial regulation at the MCS, perhaps, is to maintain a close proximity of lipidic membrane but to avoid the fusion between them. To maintain such conditions, cells need to ensure that the contact site lipids are devoid of ‘fusogens’ such as DAGs. However, the mechanisms and molecular players involved in clearing fusogens from MCS are unknown and are exciting topics for future research (Scorrano et al. 2019).

## DAG as signalling lipid in fungi – still an enigma

Although well established in animals, the role of DAGs as secondary lipid messenger has hardly been studied in fungi and the functions are still debated (Shea et al. 2006, Carrasco and Merida 2007). An early study in 1992 indicated the possible role of DAG in the cellular signalling of budding yeast. Releasing yeast cells from nitrogen starvation induces a burst of DAG and IP3 production, presumably via the phosphatidylinositol-specific phospholipase C (PI-PLC) pathway, which helps cells to escape quiescence. Interestingly, glucose-based induction of growth had no effect on DAG level. This process was shown to be under the control of Cdc25 (a protein involved in cell cycle regulation) and works parallel to the glucose-induced cyclic AMP pathway (Schomerus and Kuntzel 1992). Later, another study reported similar DAG accumulation during the G1-to-S phase cell cycle transition after nitrogen starvation (Cerbon et al. 2005). The study further confirmed that the source of this DAG pool is the sphingolipid metabolism pathway, which generally takes place in Golgi. Another study in similar line showed increased DAG level within 10–30 min of cell-cycle re-entry after G1 phase arrest using mating pheromone. Genetic analysis suggested that Cdc28 (catalytic subunit of cyclin-dependent kinase) induces such DAG production, which is followed by protein kinase C (PKC1) pathway activation (Marini et al. 1996). However, deletion of the phospholipase C (*plc1*) gene had no effect on Cdc28-mediated DAG accumulation, suggesting that the PI-PLC pathway is probably not involved in this process.

**Figure 5.**
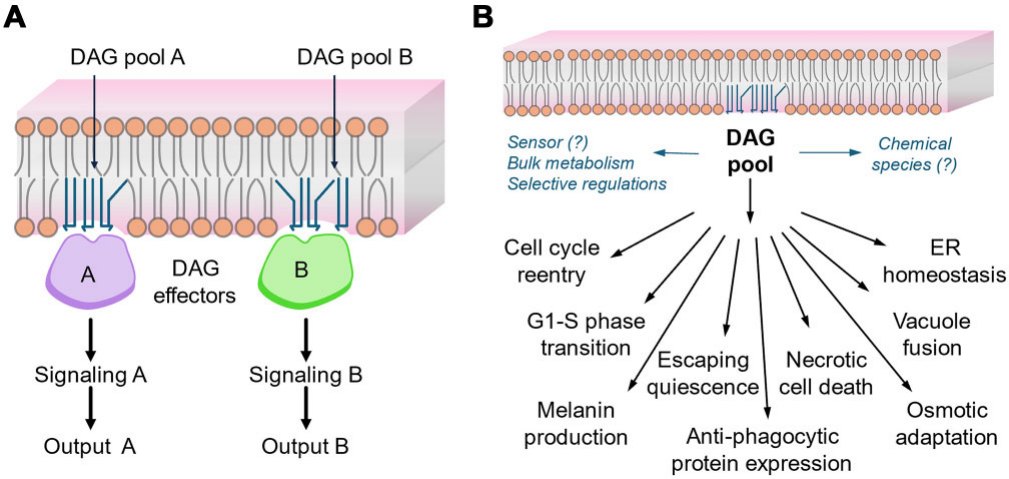
(A) DAGs work as a secondary lipid messenger by recruiting and activating C1-domain containing proteins (DAG effectors, e.g. Pkc1 in yeast), leading to cellular signalling pathway activation. However, the role of DAG in Pkc1 signalling is not well understood in fungi. Here, a proposed model showing selective DAG pool-based activation of specific signalling pathways resulting in different physiological outputs. (B) An overview of diverse physiological roles of DAG in fungi. Regulation of DAG pools in the level of chemical species by selective sensing and metabolism are the probable reasons behind such physiological outputs. However, mechanistic aspects are yet to be understood in many of the cases.

A study on Basidiomycota fungi, *Cryoptococcus neoformans*, revealed that inositol-phosphorylceramide synthase-1 (Ipc1)-mediated DAG production from sphingolipid was also involved in PKC1 pathway activation (Heung et al. 2004). However, any direct link between DAG level and putative DAG effector such as Pkc1 activation was not elucidated experimentally. For instance, an ~3-fold increase in total DAG level in ER and vacuole via *de novo* pathway did not affect the canonical localization of Pkc1, suggesting no impact on the PKC1 pathway (Li et al. 2020). Whether DAG subpopulations produced from different metabolic pathways in different subcellular locations induce distinct signalling pathways remains to be experimentally verified (Fig. 5A).

## DAG and fungal pathogenesis – a need for closer look

Fungal pathogens have come to the limelight recently as these infections have increased significantly in recent times, especially in immunocompromised patients (Fisher and Denning 2023). Lipidomic studies on several pathogenic fungi have been carried out and tried to correlate with different functions including pathogenesis and multidrug resistance (Prasad and Singh 2013, Zamith-Miranda et al. 2021). However, there is hardly any systematic study to correlate the role of specific DAGs in pathogenesis, virulence, or multidrug resistance. Interestingly, in a recent study, accumulation of 16:1 and 16:0 DAGs as well as decrease in 18:1 and 18:0-TAGs content have been observed in hospital isolates of drug-resistant *Candida auris* (NCCPF 470033) (Shahi et al. 2020). We have also reported that specific DAG subspecies (C36:0 and C36:1) regulation by Dip2 (Cmr2) in *S. cerevisiae* is crucial for adapting to different environmental stresses such as proteostasis and osmotic stress (Mondal et al. 2022). Pathogenic fungi encounter host-induced stress and need to overcome such conditions for successful infection. Also, the role of adaptation pathways such as osmoregulation has been implicated in fungal virulence (Jacob et al. 2015). This suggests an exciting correlation between DAG-mediated stress adaptation and fungal pathogenesis. In fact, *CPS1*, a member of the *DIP2* gene family, has been shown to be crucial for the virulence of pathogenic fungi such as *Cochliobolus heterostrophus, Cochliobolus victoriae, Gibberella zeae, Magnaporthe oryzae, Coccidioides posadasii*, and so on. causing severe diseases in plants and animals (Lu et al. 2003, Narra et al. 2016, Wang et al. 2016). Further progress has been made recently in this line where *cps1* knockout pathogenic fungus *C. posadasii* has been proven promising for vaccine generation (Shubitz et al. 2018).

Regulation of DAG level during infection is crucial to generate pathogenicity-related morphologies like conidia, appressorium formation, and so on. by both plant and animal fungal pathogens. Disruption of PA phosphatase (*lpp3* and *lpp5*) and *plc1* genes, which can lead to an imbalance in DAG level, has been shown to reduce or abrogate the virulence of Rice Blast Fungus, *M. oryzae* (Rho et al. 2009, Sadat et al. 2014, Zhao et al. 2022). In *C. neoformans*, DAG has been shown to be involved in melanin production via PKC1 signalling, which is an important metabolite required for its pathogenicity (Heung et al. 2004, 2005). This DAG pool has also been found to be involved in transcriptional regulation of a novel antiphagocytic protein required for the virulence of *C. neoformans* (Luberto et al. 2003, Mare et al. 2005). Interestingly, in *Malassezia spp*., causative agents of a variety of dermatological conditions, different DAG–TAG ratios were found (Celis Ramirez et al. 2020). Given such reports underlining the diverse physiological roles of DAGs, systematic studies using model fungi to correlate the spatio-temporal regulation of lipids in general and DAGs in particular will be extremely useful (Fig. 5B).

## Conclusions and future perspectives

Although the field has made significant progress in understanding DAG metabolism and homeostasis in several physiological functions, there are many intriguing aspects that need further studies. Our recent work has suggested that selective regulation of DAG pools in different subcellular sites to affect the cellular functions of yeast. Similarly, other studies have reported that the localized accumulation of the DAG pool is required for polarized cell growth, vesicle trafficking, exo or endocytosis, and cytoskeletal reorganization in yeast and animals (Baron and Malhotra 2002, Quann et al. 2009, Almena and Merida 2011, Tsai et al. 2014, Anitei et al. 2017, Ganesan et al. 2019). However, the chemical identity (chain lengths and unsaturation levels) of these DAG species is unknown. It remains to be seen if such variabilities allow DAGs to be segregated as distinct pools that are spatiotemporally distributed in a regulated manner.

Organelle-specific and localized metabolism of DAG and its subcellular functions are exciting research avenues in membrane biology. However, most of the current understanding has been derived from studies using microscopy-based localization of protein-based DAG sensors. One of the major caveats is that these cytosolic DAG sensors can access only the cytosol-facing DAG pools of organelle membranes. These are also less sensitive to the local and rare pool of DAGs. Therefore, devising and using organelle-targeted DAG sensors will provide a comprehensive understanding. Furthermore, combining click chemistry and photochemical approaches (Schuhmacher et al. 2020) with advanced imaging techniques can allow real-time observation of the DAG pool and DAG-dependent processes in living cells, providing a more dynamic and detailed picture of cellular behaviour (Uematsu and Baskin 2023, White-Mathieu and Baskin 2024). Utilization and further development of techniques such as single-molecule imaging and tracking of lipid molecules can help in targeting subcellular sites across different physiological states as well as pathogenic conditions (Adhikari et al. 2019). Many physiological processes implicated in fungal virulence and drug resistance governed by the membrane proteome are under the influence of bioactive lipids such as DAGs. Therefore, modulating the level of DAGs in the membrane could potentially be utilized for activating or deactivating such membrane-bound or associated proteins and their related downstream processes. This also broadens the scope for exploring proteins involved in DAG metabolism and regulation as potential drug targets for treating fungal diseases.
